# Clinicoradiopathologic Analysis of Odontomas: A Retrospective Study of 242 Cases

**DOI:** 10.3390/dj11110253

**Published:** 2023-10-30

**Authors:** Katherine A. DeColibus, D. Shane Rasner, Osariemen Okhuaihesuyi, Adepitan A. Owosho

**Affiliations:** 1 Department of Diagnostic Sciences, College of Dentistry, The University of Tennessee Health Science Center, 875 Union Avenue, Memphis, TN 38163, USA; kdecolib@uthsc.edu (K.A.D.); drasner@uthsc.edu (D.S.R.); 2 Missouri School of Dentistry and Oral Health, A. T. Still University, Kirksville, MO 63501, USA; ookhuaihesuyi@atsu.edu; 3 Department of Otolaryngology—Head and Neck Surgery, College of Dentistry, The University of Tennessee Health Science Center, Memphis, TN 38163, USA

**Keywords:** compound odontoma, complex odontoma, ameloblastic fibro-odontoma, jaw

## Abstract

Odontomas are considered hamartomatous lesions and are one of the two most common odontogenic tumors of the jaw. Odontomas are classified as compound or complex. Recently, ameloblastic fibro-odontoma (AFO) and ameloblastic fibro-dentinoma were reclassified as developing odontomas. Though clinically odontomas are usually asymptomatic, they have adverse effects on adjacent teeth such as tooth impaction, delayed eruption, displacement of teeth, over-retention of teeth, and can give rise to odontogenic cysts within the jaw. We sought to evaluate the clinicoradiopathologic presentations of odontomas by collecting and analyzing the clinical, radiographic, and pathologic data of odontomas diagnosed in our institution from 2013 to 2022. Over this 10-year period, there were 242 patients with a histopathological and/or radiographic diagnosis of odontoma. There was no gender predilection and ages ranged from 3 to 101 years (median, 14 years). The second decade of life was the most prevalent (57.4%). There was no jaw predilection; however, the anterior jaw was the most common location. Ninety-four (38.8%) cases presented with clinical findings. The most common finding was tooth impaction (*n* = 83). Nine (3.7%) cases were histopathologically confirmed to be associated with other lesions such as dentigerous cysts (*n* = 8) and nasopalatine duct cyst (*n* = 1). The median age (25 years) of patients diagnosed with odontomas associated with cysts was older than patients with odontomas (14 years) without associated cysts. Compound odontomas were the most common type of odontoma compared to complex and AFOs with 71.4%, 26.6%, and 2%, respectively. The majority of compound odontomas involved the anterior jaw (69.3%) and mandible (54.9%) while the majority of complex odontomas involved the posterior jaw (59.6%) and maxilla (54.7%). The four AFOs were in the posterior jaw and 75% involved the maxilla. The median age (12 years) of patients diagnosed with AFO was the youngest compared to patients diagnosed with compound (13 years) and complex (16 years). In conclusion, we analyzed the clinical, radiographic, and pathologic features of 242 new cases of odontomas. Our study reaffirms that odontomas frequently affect the pediatric population and can disrupt their dentition. Based on the result of this study, our clinical recommendation to prevent problems to adjacent teeth from odontomas is for dentists to be apt in the diagnose of odontomas to ensure that they are surgically removed in a timely manner.

## 1. Introduction

Odontomas are the most common or second most common (depending on the study) odontogenic lesion of the jaw after ameloblastoma [1,2,3,4,5,6,7,8,9,10]. Odontomas are odontogenic hamartomas [11]. Histopathologically, they are mixed epithelial and ectomesenchymal dental hard and soft tissue. The precise etiology of odontomas is unknown; however, the activation of the WNT/beta-catenin pathway has been implicated in their etiopathogenesis [12]. Odontomas are known to be associated with certain syndromes such as Gardner/familial adenomatous polyposis, Noonan, and Hermann [13,14].

Odontomas are classified as compound or complex [11]. Compound odontomas are odontogenic hamartomas in which all the dental hard and soft tissue are present in an orderly pattern resembling small tooth-like structures. In contrast, complex odontomas present with the dental hard and soft tissue in a disorderly pattern. Lesions that were previously diagnosed as ameloblastic fibro-odontoma and ameloblastic fibro-dentinoma were recently reclassified as developing odontomas [11,15].

Clinically, odontomas are usually asymptomatic, slow growing, and located in the tooth-bearing area of the jaw. They are usually diagnosed in the pediatric age group with a predilection for the second decade of life [5,9,10,16,17,18]. This is when radiographs are typically taken to investigate delayed eruption, impacted, and over-retained teeth. Odontomas can also present as an incidental finding during routine dental radiographic examination. There is no gender predilection [16,19,20,21]. The permanent dentition is more frequently involved than the deciduous dentition [19,21,22]. Radiographically, odontomas manifest as a dense radiopacity surrounded by radiolucent rim with a corticated border with the sizes usually ranging 1–2 cm in diameter; however, lesions up to 7 cm in size have been reported [23]. Odontomas may continue to enlarge or cause problems to the adjacent teeth; therefore, surgical removal is the treatment of choice for large odontomas [24,25,26,27]. Depending on the size of the odontoma, various surgical techniques such as removal of cortical plate, segmental osteotomy via extraoral excision, and sagittal split osteotomy may be necessary [24,25,26]. The surgical defect after excisional removal may be reconstructed by grafting and plating [27]. 

Odontomas are known to affect adjacent teeth by causing delayed eruption, impaction, displacement, over-retention, root resorption, widening of follicular space from retention, and subsequently can give rise to a dentigerous cyst [16,18,19,28,29,30,31]. Also, odontomas have been reported to be associated with other odontogenic lesions such as calcifying odontogenic cysts and dentinogenic ghost cell tumors [31,32]. This study aims to analyze the clinical, radiographic, and pathologic features of a relatively large series of odontomas from a single institution. 

## 2. Materials and Methods

A 10-year retrospective analysis was performed by retrieving the clinical, radiographic, and pathologic records of patients diagnosed with odontomas from the electronic health record of the University of Tennessee Health Science Center (UTHSC), College of Dentistry, from 1 January 2013 to 31 December 2022. The study was approved by the UTHSC IRB #(23-09422-XM). To confirm the diagnoses, radiographs (periapical, full mouth series and panoramic) or pathology reports of all patients with the term “odontoma” were evaluated using the WHO classification criteria. The radiographs were made for baseline, diagnosis, and treatment planning as the patients initially entered our dental school clinical program. Pathology reports were of biopsy specimens from the dental school and outside dental clinicians. All clinical charts, radiographs, and pathology reports were reviewed by authors KAD, DSR, and AAO. Only cases with confirmed radiographs and/or pathology reports were included in the study. The following clinical information was retrieved and analyzed: age at diagnosis, sex, location (maxilla vs. mandible; anterior vs. posterior), odontoma type (compound vs. complex), pathologically confirmed associated lesion, and presenting symptoms. Anterior jaw was defined as the canine-to-canine region and the posterior jaw as any area posterior to the canine.

## 3. Results

### 3.1. Clinical, Radiographic, and Pathologic Characteristics

**Figure 1:**
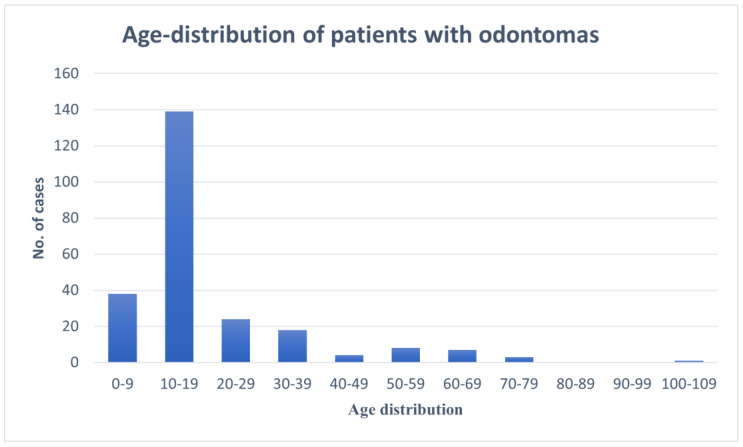
Age-distribution of patients diagnosed with odontomas.

Between 1 January 2013 and 31 December 2022, there were 360 patient records with the term “odontoma” noted. From that group, after radiographs and pathology reports were evaluated. 242 patients were confirmed to have a diagnosis of odontoma (238 odontomas and 4 ameloblastic fibro-odontomas (AFO). All cases were identified on radiographs either by the authors or outside contributors, and 200 of them were histopathologically evaluated. There were 126 (52.1%) male and 116 (47.9%) female patients. The ages ranged from 3 to 101 years, with a median of 14 years. The pediatric (<18 years old) age group made up 69.8%, and the adult age group made up 30.2%. Odontomas were most prevalent (57.4%) in the second decade of life (Figure 1), followed by the first decade of life with 15.7%, with 122 (50.4%) being identified in patients with permanent dentition, 118 (48.8%) identified in patients with mixed dentition, and only 2 (0.8%) identified in patients with primary dentition.

There were 122 (50.4%) cases of odontoma in the mandible and 117 (48.3%) cases in the maxilla. The anterior jaw was involved in 137 (56.6%) odontoma cases and the posterior jaw in 95 (39.3%) cases. Clinical findings were identified in 94 (38.8%), including impacted teeth (83 cases), delayed eruption (8 cases), retained primary tooth (1 case), partially erupted tooth (1 case), and toothache (1 case). The remaining 148 (61.2%) cases were asymptomatic and incidentally identified. A summary of the clinicoradiopathologic characteristics of odontomas are presented in Table 1. 

**Figure 2:**
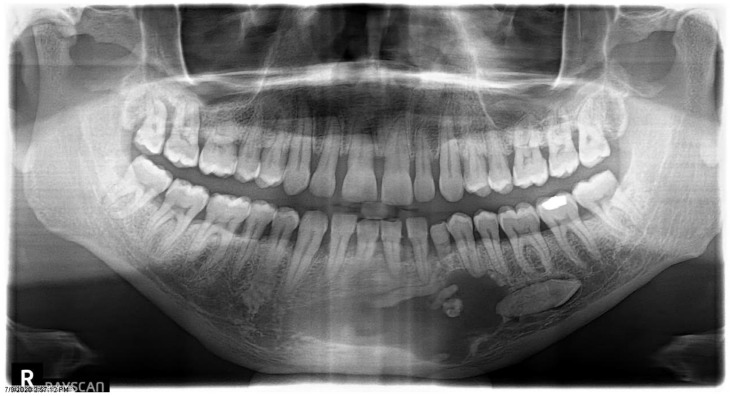
Panoramic radiograph of a 44-year-old male patient presenting with a retained primary canine shows an impacted canine, compound odontoma, and radiolucency histopathologically diagnosed as a dentigerous cyst.

Nine (3.7%) cases were histopathologically confirmed to be associated with other lesions such as dentigerous cysts (*n* = 8) and nasopalatine duct cysts (*n* = 1) (Table 2) (Figure 2). These were identified in the maxilla (four anterior and one posterior) and in the mandible (one anterior and three posterior). The male:female was 2:1 with ages ranging from 11 to 101 years (median = 25 years). Five out of the nine cases were associated with an impacted tooth, and in one case a symptom of toothache was reported.

### 3.2. Types of Odontoma

**Figure 3:**
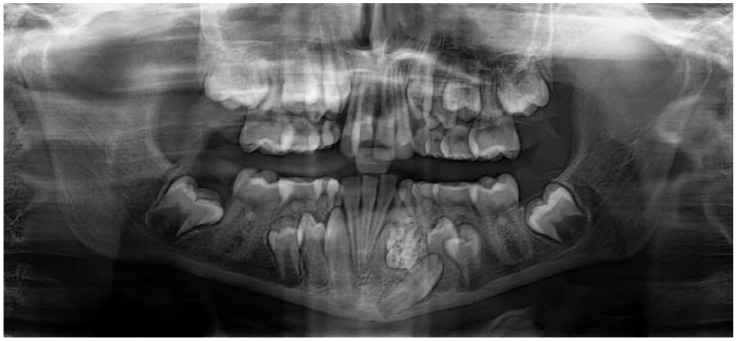
Panoramic radiograph of a 10-year-old female patient showing a compound odontoma displacing the canine tooth.

**Figure 4:**
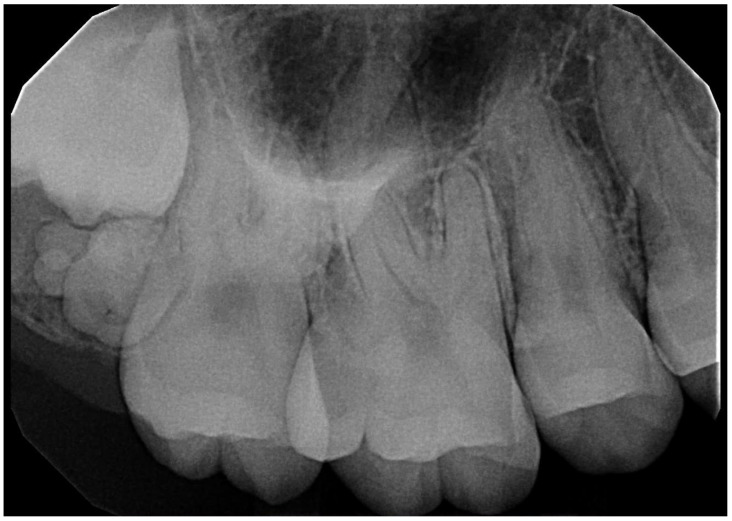
Periapical radiograph of a 20-year-old female patient showing a compound odontoma along the path of eruption of the third maxillary molar.

**Figure 5:**
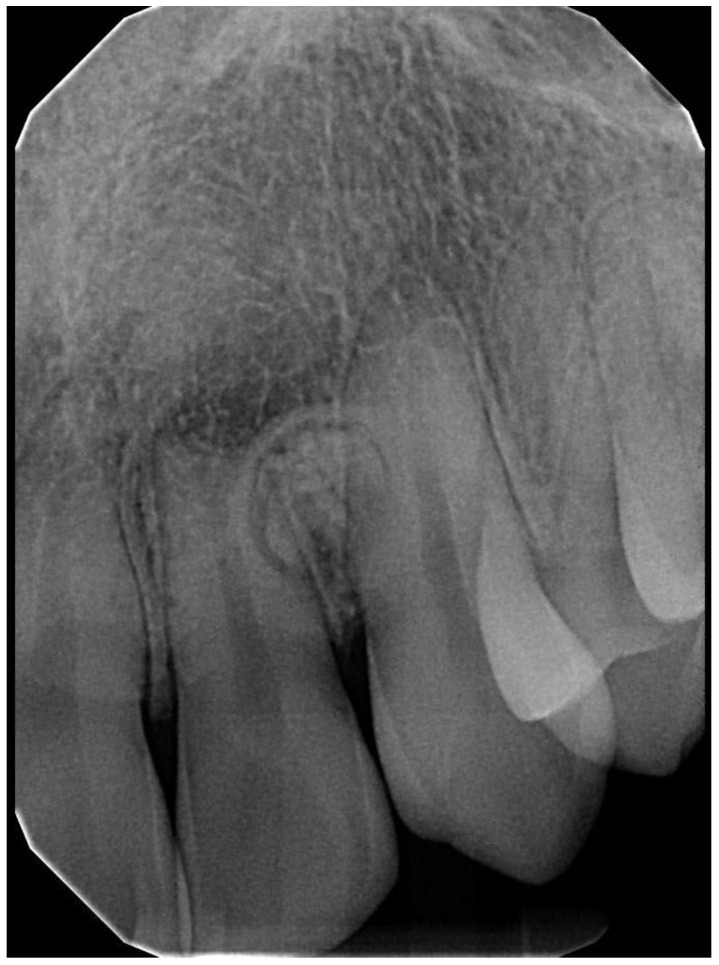
Periapical radiograph of a 21-year-old male patient showing a complex odontoma between maxillary lateral incisor and canine.

**Figure 6:**
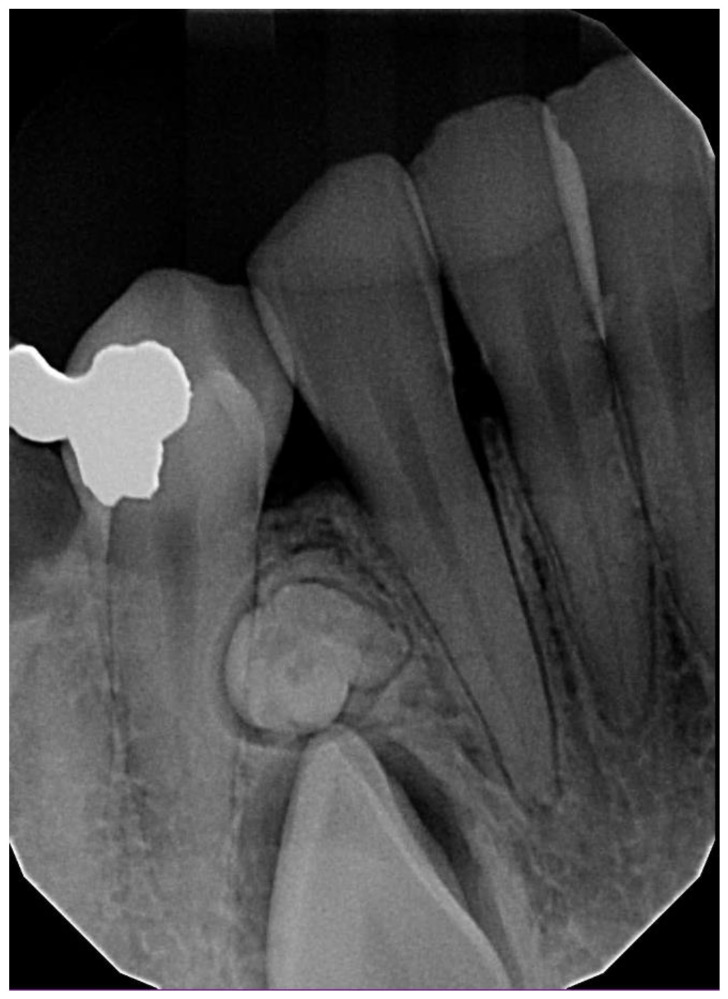
Periapical radiograph of a 29-year-old male patient showing a complex odontoma between mandibular lateral incisor and premolar causing an impaction of the canine.

After reviewing the charts (clinical, radiographic, and pathologic) only 199 of the 242 cases could be classified as compound, complex, or ameloblastic fibro-odontomas. The majority (82.6%) of our cases were histopathologically diagnosed: 45 cases had in-house radiographs to review, and 3 cases had both radiographs and histopathologic reports. Forty-three odontomas could not be further classified as either compound or complex because the histolopathology report of 37 cases did not specify, and 6 cases with radiographs alone could not also be classified as either compound or complex. In our study, compound odontomas comprised most of the cases, with 142/199 (71.4%) of the patients (Figure 3 and Figure 4). This was followed by complex odontomas, with 53/199 (26.6%) of the patients (Figure 5 and Figure 6), and AFO, with 4/199 (2%) patients. 

#### 3.2.1. Compound Odontoma

There were 76 (53.5%) male and 66 (46.5%) female patients. The ages ranged from 3 to 78 years, with a median of 13 years. Within the jaw, there were 78 (54.9%) cases of compound odontoma in the mandible, 62 (43.7%) cases in the maxilla, and 2 cases unspecified. The majority of compound odontomas involving the anterior jaw was 97/140 (69.3%) of the cases. The posterior jaw was involved in 38/140 (27.1%) of the cases, and 5 cases were unspecified. There was a prevalence for the anterior region in both the mandible and maxilla, with 69.7% and 74.6%, respectively. Clinical findings were identified in 62 (43.7%) cases of compound odontoma including impacted teeth (55 cases), delayed eruption (5 cases), retained primary tooth (1 case), and partially erupted tooth (1 case). A summary of the clinicoradiopathologic characteristics of compound odontomas are presented in Table 3.

#### 3.2.2. Complex Odontoma

There were 31 (58.5%) female and 22 (41.5%) male patients. The ages ranged from 7 to 68 years, with a median of 16 years. Within the jaw, there were 29 (54.7%) cases of complex odontoma in the maxilla, 23 (43.4%) cases in the mandible, and 1 case unspecified. The majority of complex odontomas involved the posterior jaw of 31/52 (59.6%) of the cases, the anterior jaw was involved in 20/52 (38.5%) of the cases, and 2 cases unspecified. There was a prevalence for the posterior regions in both the mandible and maxilla, with the posterior regions being 68.2% and 55.2%, respectively. Clinical findings were identified in 16 (30.2%) cases of complex odontoma: impacted teeth (14 cases) and delayed eruption (2 cases). A summary of the clinicoradiopathologic characteristics of complex odontomas are presented in Table 3.

#### 3.2.3. Ameloblastic Fibro-Odontoma

There were three (75%) male patients and one (25%) female patient. The ages ranged from 10 to 13 years, with a median of 12 years. Within the jaw, there were 3 (75%) cases of ameloblastic fibro-odontoma in the maxilla and 1 (25%) case in the mandible. All cases of ameloblastic fibro-odontomas involved the posterior jaw. A clinical finding was identified in a case presenting with an impacted tooth and a case of ameloblastic fibro-odontoma recurred. A summary of the clinicoradiopathologic characteristics of ameloblastic fibro-odontomas are presented in Table 3.

## 4. Discussion

Odontomas are hamartomatous lesions. Depending on the origin of the large series odontogenic tumor study, odontomas are considered the most common or second most common odontogenic tumor of the jaw after ameloblastoma. Studies from the United States, Canada, Greece, and Japan have reported odontoma as the most common odontogenic tumor [2,3,4,5], while studies from Nigeria, India, Malaysia, Sri Lanka, and Turkey report ameloblastoma to be the most common odontogenic tumor of the jaw [6,7,8,9,10]. These differences are attributed to the fact that most odontomas are asymptomatic and are mainly identified incidentally during clinical and radiographic examinations. Also, not all excised, biopsied odontoma specimens are submitted for histopathologic evaluation, resulting in the under-reporting of odontomas [33].

The 2017 and 2022 WHO classification of head and neck tumors reclassified AFO as a developing odontoma [11,12,13,14,15], even though these entities have a different molecular signature: *BRAF* p. V600E mutation [34]. This mutation is also present in ameloblastoma, ameloblastic fibroma, and ameloblastic fibrosarcoma but not in odontomas [34]. This suggests that this entity is molecularly distinct from odontomas. In addition, some cases of AFO are associated with locally aggressive biologic behavior and recurrence, which is a feature rarely seen in odontomas [18,35,36]. One of the four cases of AFO in this study did recur. In support of considering AFO as a developing odontoma, patients with AFO have been shown to be younger in age than patients with either compound or complex odontomas. In this study, the median age of patients diagnosed with AFO were the youngest compared to patients diagnosed with compound and complex odontomas. However, odontomas (*n* = 38) diagnosed in patients in the first decade of life in our current study did not histopathologically look like AFO. 

Using our institution’s electronic health records from 2013 to 2022, we conducted a clinicoradiopathologic analysis of 242 odontoma cases. The results of our analysis are compatible with the published literature. Similar to studies reported by Kokubun et al., Servato et al., Siriwardena et al., and Soluk-Tekkesin et al., males were slightly more affected than females [5,9,10,37]. Based on our study, odontomas frequently affected the pediatric population with a predilection for patients in the second decade of life, as reported in other studies [5,9,10,16,18,38,39]. The studies by Kokubun et al. and Servato et al. support our study that the anterior region of the jaw was more involved than the posterior [5,37]. However, the studies by Siriwardena et al., and Soluk-Tekkesin et al. reported the posterior region as the more common location [9,10]. Compound odontoma was the most common type in this present study, similar to the studies by Owens et al. [17] and Yu-Fong Chang et al. [18] but in contrast to studies by Soluk-Tekkesin et al., Fernandes et al., Nguyen and Huynh, and Tamme et al. [10,28,38,40], where complex odontoma was the most common type. The patients diagnosed with compound odontomas were younger than patients diagnosed with complex, as similarly reported by Tawfik et al., Fernandes et al., Nguyen and Huynh, and Tamme et al. [28,38,40,41]. In this study, the majority of compound odontomas involved the anterior jaw and mandible, while the majority of complex odontomas involved the posterior jaw and maxilla. The studies by Soluk-Tekkesin et al. reported that most compound odontomas were observed in the anterior maxilla, whereas most complex odontomas were found in the posterior mandible [10,16]. The study by Nguyen and Huynh identified that compound odontomas were most often located in the anterior region of both the mandible and maxilla, whereas complex odontomas were more frequently located in the posterior region of the mandible [28].

Odontomas are known to affect adjacent teeth by causing clinicoradiographic findings such as delayed eruption, impaction, tooth/root displacement, over-retention, root resorption, and widening of follicular space from retention, which subsequently can give rise to a dentigerous cyst. The study by Nguyen and Huynh reported that 58% of odontomas were associated with adjacent tooth eruption disturbance and 48% of odontomas were associated with tooth/root displacement [28]. The study by An et al. identified that 62% of odontomas were associated with impaction of permanent teeth and about 30% of the odontomas were associated with tooth displacement [19]. The study by Katz reported that 41% of 396 odontomas were associated with adjacent tooth eruption disturbance [29]. In our study, 34.3% (83/242) were associated with impaction of an adjacent tooth, and 3.7% (9/242) were associated with adjacent tooth eruption disturbance. The studies by Nguyen and Huynh, and Bereket et al. identified that compound odontomas (75% and 69%, respectively) were more prone to cause adjacent tooth eruption disturbance compared to complex odontomas (41.3% and 50%, respectively) [28,30]. The same trend was identified in our study with 43.6% (62/142) of compound odontomas vs. 30.2% (16/53) of complex odontomas. The study by Soluk-Tekkesin et al. found that 7.5% (12/160) of odontomas were associated with odontogenic cysts [16]. The study by Yu-Fong Chang et al. reported that 9% of odontomas were associated with dentigerous cysts [18]. In our current study, we identified 3.7% (9/242) associated with cysts (dentigerous and nasopalatine duct). Our study showed that the median age (25 years) of patients diagnosed with odontomas associated with cysts was older than patients with odontomas (14 years) without associated cysts. This reflects the time required for the widening of follicular space from tooth retention, which can subsequently give rise to a cyst.

## 5. Conclusions

We analyzed the clinical, radiographic, and pathologic features of 242 new cases of odontomas. Our study reaffirms that odontomas frequently affect the pediatric population and might cause problems to adjacent teeth. Based on the result of this study, our clinical recommendation to prevent these problems is for dentists be apt in the diagnose of odontomas to ensure that they are surgically removed in a timely manner.

## Figures and Tables

**Table 1:** A summary of the clinicoradiopathologic features of odontomas.

Number of Cases	242
Gender	
Male	126
Female	116
Age (Years)	
Range	3–101
Mean	19.5
Median	14
Adult/Pediatric	
Pediatric (<18 years)	169
Adult (>18 years)	73
Type of Dentition	
Primary (<6 years)	2
Mixed (6–13 years)	118
Permanent (>13 years)	122
Age Distribution (Years)	
0–9	38
10–19	139
20–29	24
30–39	18
40–49	4
50–59	8
60–69	7
70–79	3
80–89	0
90–99	0
100–109	1
Jaw	
Maxilla	117
Mandible	122
Unspecified	3
Location	
Anterior	137
Posterior	95
Unspecified	10
Type	
Compound	142
Complex	53
AFO	4
Unspecified (odontoma)	43
Pathologic/radiographic confirmation	
Pathologic alone	197
Radiographic alone	42
Both	3
Associated Cyst	
Dentigerous	8
Nasopalatine duct cyst	1
Clinical Findings	
Impacted	83
Delayed eruption	8
Retained primary	1
Partially erupted	1
Toothache	1

**Table 2:** The clinical characteristics of patients diagnosed with odontomas associated with cyst.

Case No.	Age	Sex	Type	Location	Clinical Findings
1	13	F	AFO	Maxilla—Posterior	NR
2	25	M	Complex	Mandible—Posterior	Toothache
3	31	M	Odontoma (U)	Maxilla—Anterior	NR
4	11	M	Compound	Maxilla—Anterior	Impacted #27
5	44	M	Compound	Mandible—Anterior	Impacted #22
6	13	F	Odontoma (U)	Maxilla—Anterior	Impacted #11
7	13	M	Odontoma (U)	Maxilla—Anterior	Impacted #6
8	26	M	Odontoma (U)	Mandible—Posterior	Impacted #17
9	101	F	Odontoma (U)	Mandible—Posterior	NR

F—female, M—male, U—unspecified, AFO—ameloblastic fibro-odontoma, NR—not reported.

**Table 3:** A summary of the clinicoradiopathologic features of compound, complex, and ameloblastic fibro-odontomas.

Type	Compound	Complex	AFO
No. of cases	142	53	4
Gender			
Male	76	22	3
Female	66	31	1
Jaw			
Maxilla	62	29	
*Anterior*	44	13	3
*Posterior*	15	16	-
*Unspecified*	3		3
Mandible	78	23	
*Anterior*	53	7	1
*Posterior*	23	15	-
*Unspecified*	2	1	1
Unspecified	2	1	
Age (Years)			
Range	3–78	7–68	10–13
Mean	17.7	23.7	11.75
Median	13	16	12
Pathologic/radiographic			
Pathologic alone	120	36	4
Radiographic alone	20	17	-
Both	2	-	-
Clinical Findings	55 impacted	14 impacted	1 impacted
	1 retained		
	5 delayed eruptions	2 delayed eruptions	
	1 partially erupted		

## Data Availability

The data are unavailable due to privacy or ethical restrictions. Please contact the corresponding author.
